# Pattern Recognition of Neurotransmitters: Complexity Reduction for Serotonin and Dopamine

**DOI:** 10.3390/bios15040209

**Published:** 2025-03-25

**Authors:** Ibrahim Moubarak Nchouwat Ndumgouo, Emily Devoe, Silvana Andreescu, Stephanie Schuckers

**Affiliations:** 1 Department of Electrical and Computer Engineering, Clarkson University, Potsdam, NY 13699, USA; sschucke@clarkson.edu; 2 Department of Chemistry and Biomolecular Science, Clarkson University, Potsdam, NY 13699, USA; devoeel@clarkson.edu (E.D.); eandrees@clarkson.edu (S.A.)

**Keywords:** machine learning, pattern recognition, neurotransmitters, biosensors, voltammetry

## Abstract

In this work, we simultaneously detected and predicted the concentration levels of serotonin (SE) and dopamine (DA) neurotransmitters (NTs) for in vitro mixtures, with measurements obtained using conventional glassy carbon electrodes (CGCEs) and differential pulse voltammetry (DPV). The NTs were estimated by deconvolving the multiplexed signals of both NTs using Principal Component Analysis with Gaussian Process Regression (PCA-GPR) and Partial Least Squares with Gaussian Process Regression (PLS-GPR), both with exponential–isotropic kernels. The average testing accuracies of estimation using PCA-GPR for DA alone, SE alone and their mixture (DA–SE) were 87.6%, 88.1%, and 96.7%, respectively. Using PLS-GPR, the testing accuracies of estimation for DA alone, SE alone, and their mixture (DA–SE) were 87.3%, 83.8%, and 95.1%, respectively. Furthermore, we explored methods of reducing the procedural complexity in estimating the NTs by finding reduced subsets of features for accurately detecting and predicting their concentrations. The reduced subsets of features found in the oxidation potential windows of the NTs improved the testing accuracy of the estimation of DA–SE to 97.4%. We thus believe that reducing complexity has the potential to increase the detection and prediction accuracies of NT measurements for practical clinical uses such as deep brain stimulation.

## 1. Introduction

Neurotransmitters (NTs) including dopamine (DA), serotonin (SE), glutamate (GT) and epinephrine (EP) play crucial roles of ensuring the body’s physical and mental health by taking part in neurological impulse transmissions in both the central and peripheral nervous systems [1,2,3,4,5,6]. They coexist in the cerebrospinal fluid mainly in the brain and other biological fluids like saliva, urine, etc. They act as biomarkers for some neurological diseases, and disruption of their types and/or equilibrium concentration levels has been shown to cause neurological disorders [1,2,3,4,5,6]. For instance, a deficiency in DA is hypothesized to cause Parkinson’s disease (PD) and Alzheimer’s disease [1,2,3,4,5], whereas a deficiency in GT can cause schizophrenia [7]. Therefore, the accurate, simultaneous, and real-time detection and prediction of the concentration levels of individual NTs in the complex brain environment are essential for the early diagnosis and treatment of these neurological diseases [1,2,3,4,5,6,7]. Deep brain stimulation, a promising therapy for PD, requires the fast and accurate detection and monitoring of NTs [8,9,10]. However, this is very challenging because NTs are produced and secreted at very low and constantly varying levels as well as perform their neurologic functions quickly and have short lifespans [11] These make the design of accurate, sensitive, selective, and reliable systems for the in vivo detection and real-time monitoring of NTs difficult [1,2,3,4,5,12,13,14,15]. Furthermore, some NTs have similar interaction effects on sensors, so that it is difficult to create a sensor that is both sensitive as well as selective (specific) for their detection. Attempts have been made to develop fast, sensitive, accurate, specific, less-invasive, and low-cost detection systems for specific NTs or groups of NTs. Chromatography and capillary electrophoresis methods have long been used to simultaneously detect NTs. However, these techniques are slow, less sensitive, complex, and not appropriate for the in situ real-time detection of NTs [16]. New approaches for simultaneous detection based on the electronic nose (e-nose) or electronic tongue (e-tongue) [17] as well as microdialysis [18] are being explored. These measurement techniques have the advantages of being less invasive and more accurate, even for multiple analytes, and are stable for long durations under use. However, they have a low temporal resolution [11]. To mitigate this limitation, electrochemistry has been used to directly measure some NTs like the ones we studied (DA and SE) using voltammetry. Voltammetry techniques like differential pulse voltammetry (DPV) and fast scan cyclic voltammetry (FSCV) have the potential to mitigate the temporal resolution problem faced by the other measurement techniques and minimize the measurement of background (charging) current [1]. Their drawback is the inability to efficiently deconvolve the “true” signals of individual NTs from the multiplexed signals from the different NTs captured by the sensors. This results in crosstalk between the patterns of different measured NTs when simultaneously recorded by sensors at certain potentials [1]. Thus, in this work, we developed predictive machine learning models based on pattern recognition (PR) techniques to simultaneously detect and predict the concentration levels of DA and SE with DPV measurements. Additionally, we determined reduced-feature subsets, which have the potential to simultaneously estimate NTs with higher accuracies. The PR techniques proposed here are Principal Component Analysis with Gaussian Process Regression (PCA-GPR) and Partial Least Squares with Gaussian Process Regression (PLS-GPR), which are the modified forms of prior pattern recognition techniques, namely, principal component regression (PCR) and Partial Least Squares regression (PLSR), respectively. The traditional PCR and PLSR apply linear regression and various kernels, with the radial basis function (RBF) being the commonly used kernel.

We combined Principal Component Analysis (PCA) and Partial Least Squares (PLS) with the proposed Gaussian processes regression technique (GPR) to overcome the limitations faced by linear regression in the analyses of small and potentially noisy datasets with a multidimensional input space. GPR is a regression technique with nonparametric and nonlinear properties. It is based on Bayesian probability theory and hence is suitable for the controlled, explicit, and quantitative interpolation of data points scattered in a high-dimensional input space. It is robust to noise, tolerant to uncertainty in the data, and provides performance comparative to an infinite-width neural network [19]. In GPR, the estimate $\tilde{y}$(*x*) of a continuous, regular, and smooth function y(*x*) is established at arbitrary locations *x* together with the quantification of uncertainty and expected errors. y(*x*) takes a d-dimensional vector input x and maps it to a single response having an unknow form. Since the single response is unknown, a dataset of N independent observations y(n) (considered to be samples of y(*x*)) with values at *x*(n) is made and used for the prediction of y(*x*). Equation (1) shows the applied function–space view for GPR.

(1) $$\tilde{y}\left(x\right)=\sum_{h}^{H}w_{h}{Ø}_{h}(x)$$

where *x* is the input space, $w_{h}$ are the constant coefficients or weights, Ø_*h*_ is a set of fixed unspecified basis functions, and *H* is the number of linear combinations of the basis function.

Compared to simple linear regression, the Gaussian Process Regression technique coupled with Principal Component Analysis or Partial Least Squares was found to perform better on our dataset having 27 raw data as the input dimensional space.

The aims of this work were (1) to develop novel predictive models, Principal Component Analysis with Gaussian Process Regression and Partial Least Squares with Gaussian Process Regression, for accurately and simultaneously estimating dopamine and serotonin concentration levels in in vitro mixtures using differential pulse voltammetry with conventional glassy carbon electrodes; (2) to decrease the procedural complexity of the hypothetical system by reducing the number of scanning voltages needed for differential pulse voltammetry.

## 2. Material

### 2.1. Reagents

Dopamine hydrochloride (DA) and serotonin hydrochloride (SE) were obtained from Sigma-Aldrich (Chemie GmbH Eschenstr. 5, 82024 Taufkirchen, Germany). Combinations of these neurochemicals were made in triplicates. All the chemicals were of analytical reagent grade, and Milli-Q water was used throughout the experiments to prepare the solutions.

### 2.2. Equipment

Differential pulse voltammetry (DPV) was used with conventional glassy carbon electrodes (CGCEs) to collect the in vitro data for the NT mixtures. A neutral solution of 7.0 pH, 0.1 M PBS was maintained for the voltammograms, and the parameters of the pulse applied for the DPV had the following specifications: initial voltage = −0.16 V, final voltage = 0.88 V, increment = 0.04 V, amplitude = 0.05 V, pulse width = 0.05 s, sample-width = 0.0167 s, pulse period = 0.2 s, quiet time = 2 s and sensitivity = 1.exp(-6) ampere per volt (A/V).

### 2.3. Data Collection

**Figure 1:**
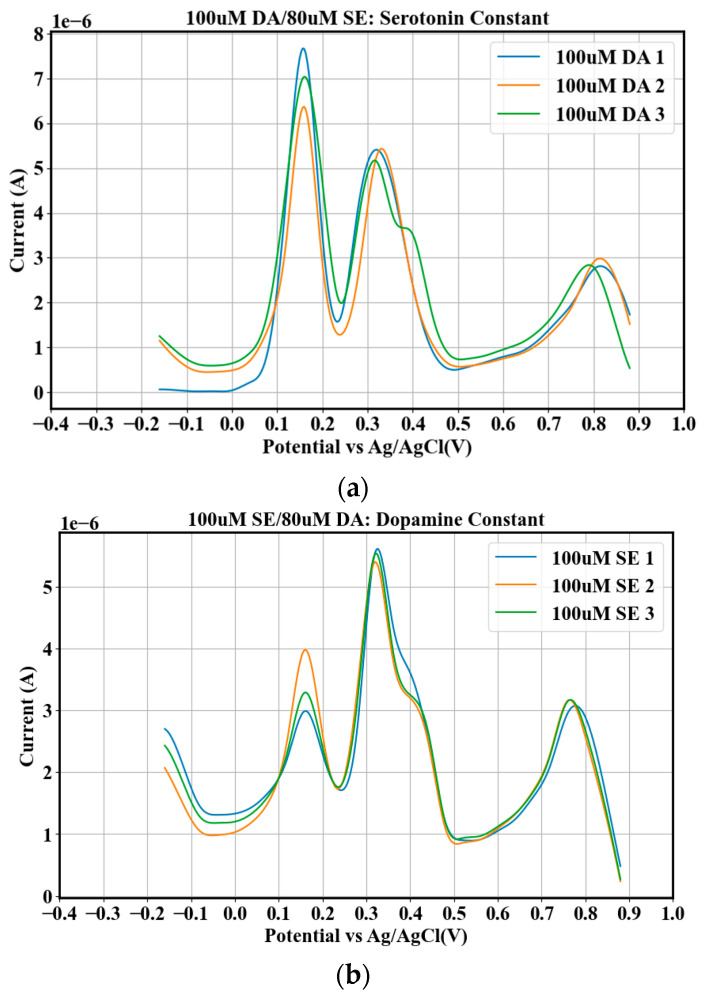
Sample voltammograms of DPV plots for three measurements of (**a**) 100 µM DA/80 µM SE at constant SE and (**b**) 100 µM SE/80 µM DA at constant DA. (**a**) More current flows in the window between 0.05 V and 0.42 V, with peaks around 0.15 V and 0.35 V representing the areas of highest responses of the NTs to the applied voltage. (**b**) Less current flows, and the window is shifted to the right between 0.1 V and 0.5 V.

We used 0.3 mm alumina to polish bare CGCEs. The bare electrodes were then dipped in distilled water and sonicated for about 5 min. Finally, distilled water was used to rinse the bare electrodes three times, followed by a final rinsing with methanol. Mixtures of NTs were prepared by combining discrete concentrations of DA and SE in vitro, with each NT concentration varying from 0 µΜ to 100 µΜ in a step of 20 µΜ, producing 6 discrete concentrations for each of the two NTs. For a given discrete concentration of DA, the six different discrete concentrations of SE were mixed with it, and the data were collected. This constituted the first dataset at a constant DA and gave a total of 6 × 6 = 36 discrete combinations (6 constant DA × 6 different SE = 36). This was repeated for SE. This constituted a second dataset at a constant SE and gave a total of 6 × 6 = 36 discrete combinations (6 constant SE × 6 different DA = 36). The total number of discrete combinations were 36 + 36 = 72. Measurements were made in triplicate for each mixture. For each of the 3 repetitions corresponding to each combination, 2 were used as training sets and 1 as the testing set, giving 144 sets for training and 72 sets for testing. Table 1 shows the distribution of the training and testing sets for the experimental repetitions. For any discrete mixture (combination), data were collected by applying a series of 27 different potentials between the working electrode and the counter electrode of the DPV starting from −0.16 V to 0.88 V versus Ag/AgCl with a step of 0.04 V and recording the corresponding currents. Scanning in the range of −0.16 V to 0.88 V was performed to ensure the stability of the CGCE, preventing it from breaking down and from being passivated over the voltage of around 1 volt. The effects of passivation/fouling of the electrodes on the data were further reduced by using different electrodes to collect data for different triplicates and regularly cleaning the electrodes after each measurement for a given triplicate. Cleaning was performed by running FSCV on PBS solution, followed DPV after each measurement for a given triplicate. These steps helped us to model the noise in the cell (blank reading). The blank readings were incorporated in the datasets to improve the robustness and generalization of the models for in vivo applications. The scanning voltages in the range between 0.16 V and 0.88 V corresponded to the original features of the data. Figure 1 shows sample voltammograms for the DPV readings of the mixtures of various DA and SE concentrations.

## 3. Methods

### 3.1. Data Preprocessing

To prevent overfitting of the models, white Gaussian noise and the background DC signal from the electronic DPV system were removed by passing the data through a detrend filter. This step was performed to prevent the target regression functions from fitting all the data points, including the noise. The procedure was enhanced by first subtracting the charging, background, and DC offset currents from the whole dataset of discrete mixtures of both NTs. This DC value was obtained by plotting the data of the current against the potentials and finding the portions of the curves where no current change was observed for consecutive five changes in potentials.

### 3.2. Experimental Implementation

PCA-GPR was applied in two folds. Firstly, Principal Components Analysis (PCA) was applied to extract and reduce the explanatory variables or feature dimensions of the original dataset (voltammetry readings) to smaller uncorrelated feature dimensions called the principal components. This was performed by reducing the correlation between the original or explanatory features and maximizing the variance between them, which was followed by applying Gaussian Process Regression to find the best fit. The principal components obtained maximized the variance for the explanatory features and not the target output or dependent variable [20], in this case, the NT concentration levels. Conversely, in PLS-GPR, latent variables were computed rather than principal components. The latent variables explained the dependent variable as well as the explanatory features due to their maximized covariances.

### 3.3. Feature Reduction

**Figure 2:**
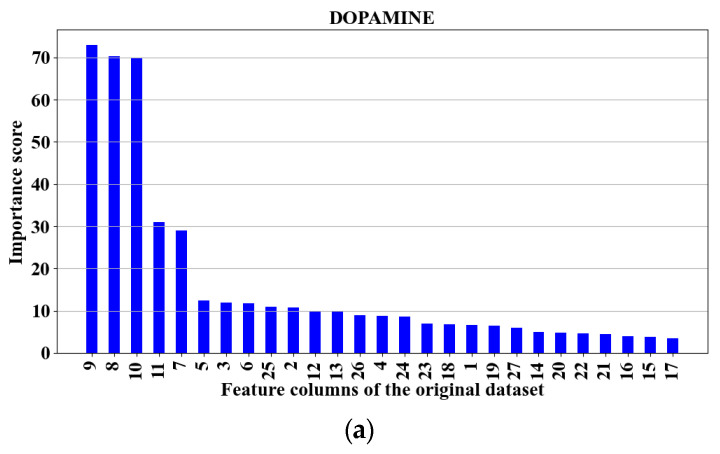

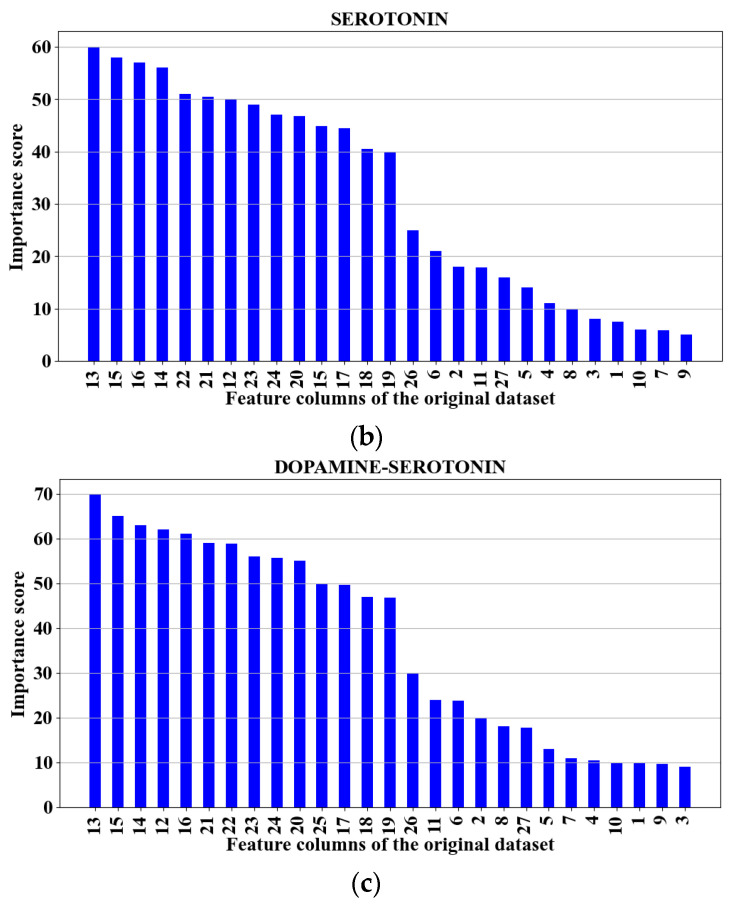
Plots of the features for the original dataset (with training sets 1 and 2 and test set 3) ranked in order of importance based on the F-test for (**a**) DA, (**b**) SE, (**c**) DA–SE. The F-test selects and ranks features by measuring the ratio of variances between multiple features.

Additionally, we explored the reduction in the number of voltages, herein called “features”, needed to achieve an accurate measurement of the DA–SE in mixtures by selecting the most representative parts corresponding to the oxidation potential windows (OPW) of the NTs. Prior work has determined the OPW for both NTs in discrete mixtures [21,22] and concluded that DA is oxidized at around 0.6 V and is reduced at around −0.1 V at fast scan rates greater than 300 V/s in anodic and cathodic sweep directions, respectively. Based on this, we approximated the OPW to correspond to the regions between 0 V and 0.52 V of the current response to the potentials from the mixtures of DA and SE. Using these features corresponding to the OPW reduced the complexity of measuring the NTs due to the fact the NTs are more responsive to potential changes within these windows and less time is taken for detection due to the reduced scanning steps required by the DPV [1]. Figure 2 shows plots of the feature columns for the original dataset with training sets 1 and 2 and test set 3, ranked in order of importance based on the F-test for dopamine, serotonin, and their mixtures.

### 3.4. Further Feature Reduction

The main idea in this study was to use as few features as possible for predictions to improve the models’ performance and reduce overfitting, as argued in prior works [23,24,25,26]. To this aim, we explored the effects of further reducing the number of features needed to accurately detect and predict the concentrations of NTs (DA and SE). Reducing the number of scanning voltages and time needed in the process reduce the overall complexity of the system. To reduce the number of possible subsets to be determined and analyzed, the intersecting features between the F-test, MRMR-test and RReliefF-test were used to rank the feature columns in their order of importance. The F-test selects and ranks features by measuring the ratio of variances between multiple features, MRMR minimizes the redundancy of the features and ranks them based on maximum relevance, while the RReliefF-test discriminates and ranks features with strong interactions. The feature subsets were found by gradually and orderly increasing the number of features starting with the most important one. We then selected the intersecting features from the three tests to represent the feature subsets that reduced the system’s complexity in the real-time measurement of DA and SE. Figure 2 shows a sample plot of the intersecting features of DA–SE. Here, three subsets of features (with four to six features each) from the 27 features, corresponding to 27 potentials from −0.16 V to 0.88 V at a step of 0.04 V, were selected as the best predictors. Table 2, Table 3 and Table 4 summarize the average training and testing accuracies as well as the R^2^ values when PCA-GPR was applied to all the features and to the reduced feature subsets. The columns of the feature subsets from the columns of the original dataset found were columns 9, 8, 10, 11 and 7 for DA, columns 13, 15, 16 and 14 for SE, and columns 13, 15, 14, 12 and 16 for DA–SE. These features are also plotted in Figure 2. The feature subsets correspond to the original scanning voltages (in volts) of [0.16, 0.12, 0.2, 0.24, 0.08], [0.32, 0.4, 0.44, 0.36], and [0.32, 0.4, 0.36, 0.28, 0.44] for DA, SE, and DA–SE, respectively.

### 3.5. Framework of the Experiments

**Figure 3:**
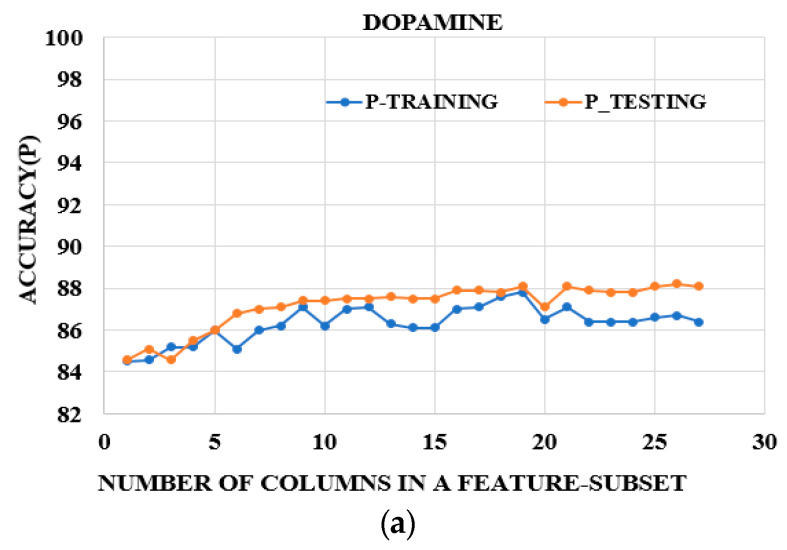

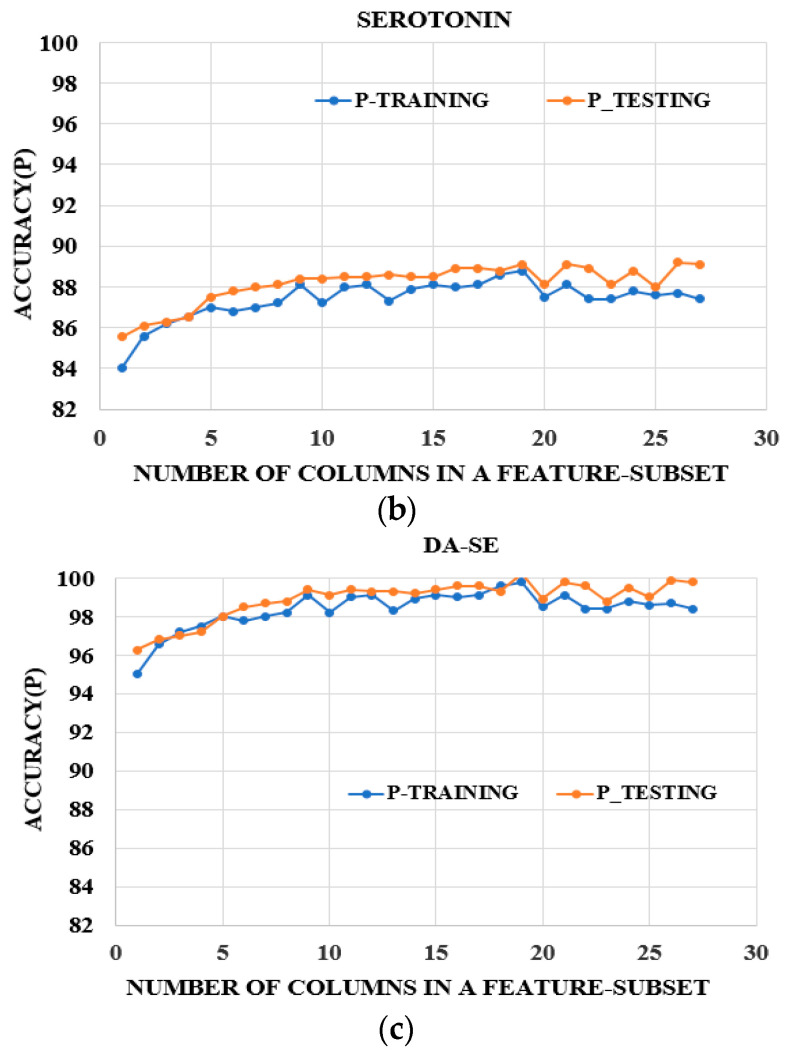
The plot for accuracies of the different feature subsets derived from the original dataset for (**a**) DA detection, (**b**) SE detection, (**c**) DA–SE detection.

**Figure 4:**
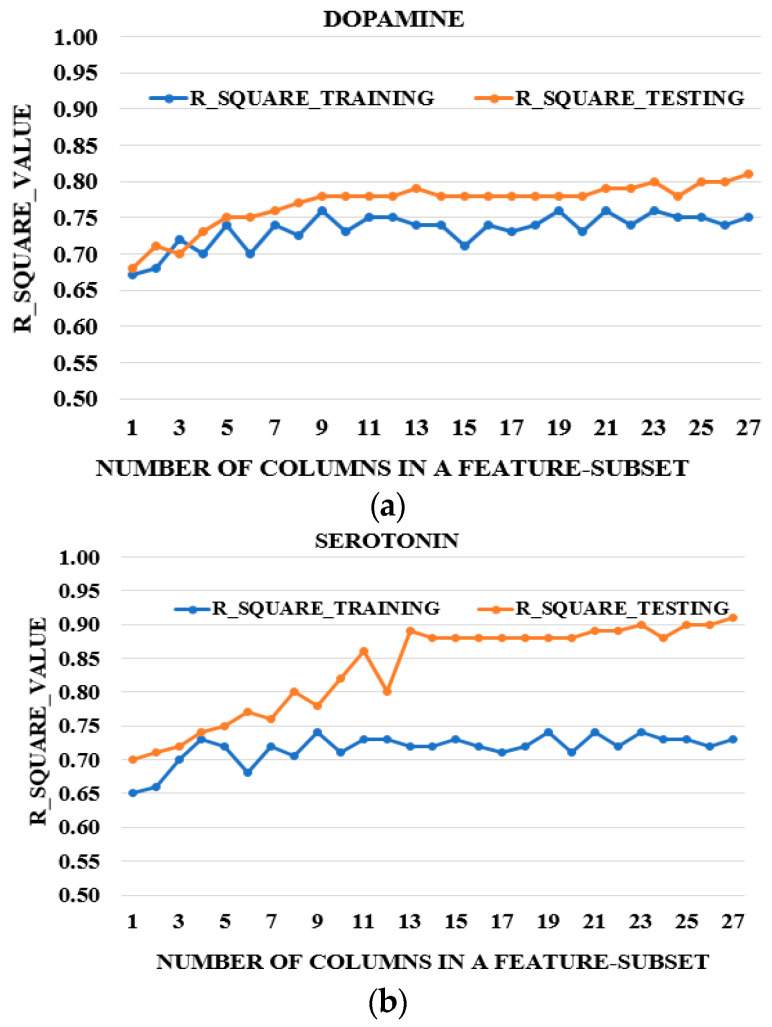

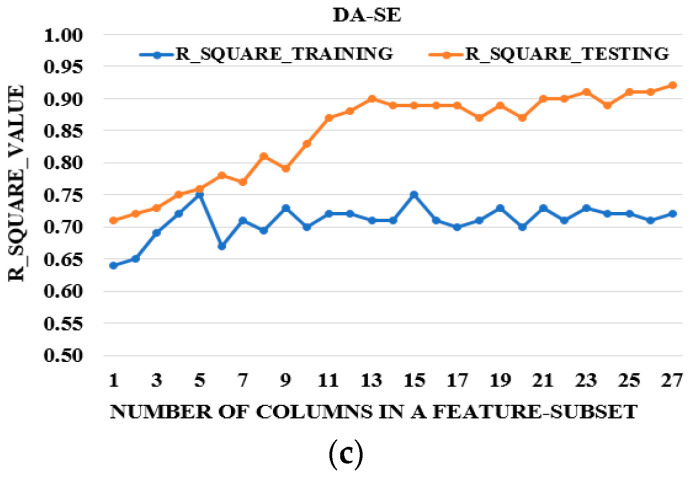
The plot of R^2^ values of the different feature subsets derived from the original dataset for (**a**) DA fitting, (**b**) SE fitting (**c**) DA–SE fitting.

We used Gaussian Process Regression with the following hyperparameters, tuned by the grid-search technique: preset = exponential GPR, basis function = constant, kernel function = exponential and isotropic, and regularization value = 20. The data were standardized using the z-norm and Bayesian optimization was applied. Average models from 10-fold cross-validation for training were computed. These average models were tested with separate independent sets. For PCA-GPR, regressions were applied on six principal components accounting for 95% of the variance, and six latent variables were computed and used as predictors for PLS-GPR. All the regressions were conducted using MATLAB-2023(b). The evaluation metrics were the accuracies computed by subtracting the mean absolute error (MAE) from 100 (Figure 3) and the R-square values (Figure 4). The plots of the accuracies in Figure 3 show similar trends for DA alone, SE alone, and their mixtures (DA–SE), as the curves for the testing accuracies lie on top of the curves for the training accuracies. These results showed that our models mitigated the effects of overfitting for the three classes of NTs. The R-square values indicated the regression fit. Each experiment was conducted three times with two sets taken as the training sets and the third set as the testing set each time, as described in Table 1. The average accuracies and R-square values for training (TR) and testing (TE) were computed for the three observations and are shown for all data and the reduced set based on the OPW for PCA-GPR (Table 2) and PLS-GPR (Table 3). The discrepancies in the R^2^ values in the training and testing experiments in the case of SE and DA–SE occurred because SE is a special derivative of a tryptophan precursor; therefore, it is not a catecholamine as it has no catechol nucleus and is less active, so SE oxidizes at higher potentials with values varying between the training and testing, as shown on Figure 4b. DA is a derivative of a phenylalanine precursor. It is a catecholamine and more active as it oxidizes at lower potentials, with constant values between training and testing (Figure 4a). Mixtures of both NTs reduce the activity of DA, causing variations in the oxidation potentials of the NT mixtures. These changes are reflected in the data recorded and the regression fitting (R^2^ values), as presented in the plots for the training and testing in Figure 4c.

## 4. Results

Table 2 presents the average results for both the accuracies and R^2^ values for the training (TR) and testing (TE) of three PCA-GPR models: (1) prediction of DA only, (2) prediction of SE alone, and (3) simultaneous prediction of DA–SE. Table 3 provides similar results for PLS-GPR. Figure 3 and Figure 4 show plots of the accuracies and R^2^ values obtained with the reduced feature subsets for DA–SE estimation, respectively.

## 5. Comparison with Other Works

DA and SE were simultaneously detected, and their concentration levels were predicted with accuracies ranging from 87.6% to 96.7%, improving upon the outcomes obtained in following works:

1. Sazanova et al. [1] used seven principal components and seven latent variables from the original data for application in the traditional pattern recognition techniques, PCR and PLSR, respectively. Their models detected and predicted the concentrations of DA alone and SE alone in in vitro mixtures. They obtained a test accuracy ranges for correct classifications of 42–62% for DA and 33–50% for SE. To increase the ranges of these accuracies, they extended the ranges for correct prediction to include one level above and below the true concentration level, obtaining new accuracy ranges of 81–91% for DA and 91–100% for SE. This extension of levels increased the confidence interval for the estimation of the NTs, deviating from the “true” concentration estimates for the NTs in the mixtures. We thus believe that simultaneously detecting and predicting the “true” concentration levels of DA and SE in vitro with the modified PCR and PLSR, i.e., PCA-GPR and PLS-GPR models respectively, yields better results than detecting and predicting the concentrations of the NTs separately.
2. Movassaghi et al. [2] applied rapid pulse voltammetry wave forms coupled with PLSR (RPV-PLSR) using a smart pulse approach for the simultaneous monitoring of DA and SE for in vitro and in vivo complex environments across time scales. The work included background current in NT monitoring and used 518 latent variables to train the PLSR models to construct their in vitro model. These steps caused overfitting, as argued by [23,24,25,26]. Further, they used a moving average kernel to map each latent variable to low, medium, or high correlation using the shading technique, ranging from 0 to 100% shading. This step assimilated the voltage-dependent current-response data to be time-series data without applying the guidelines stated by [26] for the conversion of any data to time-series data. We thus demonstrated that it is possible to select subsets of predictors to reduce the complexities and time required for NT detection and prediction, hence avoiding any conversion of the data structure by using GPR, which efficiently fills the spaces between data points through interpolation to generate continuous representations [18].

Additionally, Kim et al. [19] compared the performance of PLSR and PCR in the detection of neurochemicals with FSCV data. They showed that PLSR performed better than PCR in detecting the concentrations of neurochemicals. Similarly, we show that PLS-GPR performs better on training data than PCA-GPR, as claimed by Kim et al. [19], while PCA-GPR performs better on the test data, as claimed by Sazonova et al. [1], for true predictions. Since we used DPV to record our data, the training accuracies were of course of low interest since they could not capture the generalization ability of our proposed approach. Nevertheless, the results show that PLS-GPR is more susceptible to overfitting, with this being pronounced when detecting SE alone, while PCA-GPR is slightly susceptible to underfitting.

**Figure 5:**
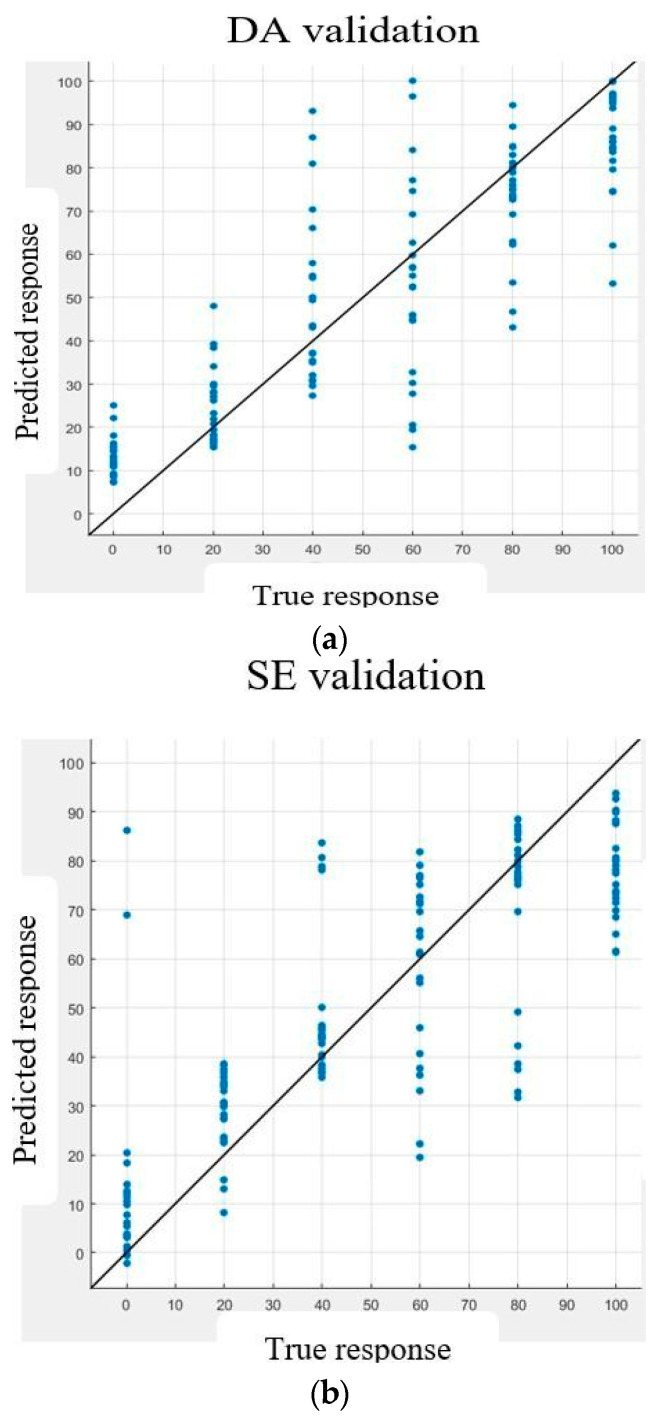

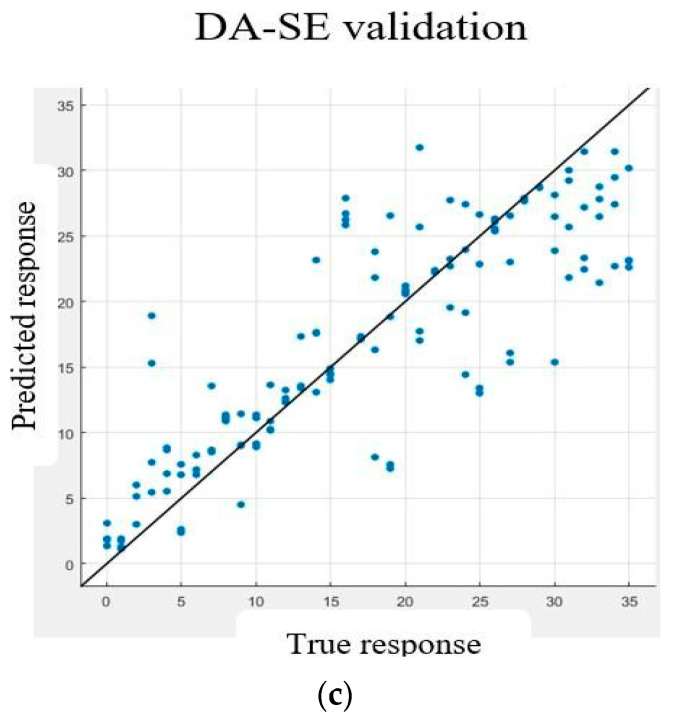
Sample validation plots with the application of PCR-GPR in Exp 1. (**a**) Prediction of DA. (**b**) Prediction of SE. (**c**) Prediction of DA–SE.

The blue dots on Figure 5 represent the observations, and the 45-degree line represents perfect predictions. In (a) and (b), the axes have labels ranging from 0 to 100, representing the concentrations of the separate NTs. In (c), the axes represent the coded labels of 0 to 36, representing the joint concentrations of NTs (e.g., 0 represents 0 µΜDA and 0 µΜ SE, while 36 represents 100 µΜDA and 100 µΜSE). In (c), the observation data points are less spread around the 45-degree line, which follows the direction of greatest variance in the observation data points, accounting for the better fit of the line and the higher accuracy.

## 6. Validation

The dataset used by Sazanova et al. [1] was used to validate our models. The assumption made prior to using their dataset was that the voltammograms of the mixtures of DA and SE with constant DA and constant SE were the same. The feature columns corresponding to the reduced feature subsets found in this work were selected from their data, and the training and testing were conducted as described herein. The results obtained are summarized in Table 5. These results are comparable to those in Table 4, with marginal discrepancies. These discrepancies could have been due to our assumption regarding the dataset, as the voltammograms did not actually match each other, as ours did, as presented in Figure 1.

## 7. Discussion

Table 2 and Table 4 regarding PCA-GPR application show improvements in accuracies: from a maximum of 96.7% for raw features to 97.4% when the features inscribed in the oxidation potential windows were used for testing the simultaneous DA–SE estimation. This could be explained by the fact that the use of fewer predictors or features led to the fast convergence of the models during training or testing and reduced the overfitting of the models, thereby yielding better results, as extensively discussed in [27]. Figure 5 shows the validation plot for DA–SE estimation using the reduced features.

For practical applications such as deep brain stimulation therapies to control diseases like Parkinson’s disease, further explorations need to be conducted to determine whether the models generalize to “in vivo” experiments. Therefore, additional experiments and factors should be considered prior to practical implementation. Firstly, the passivation of the sensors, which greatly contributes to the reduction in performance, should be analyzed. This is due to the reduction in the sensitivity of the sensors when a particular NT is adsorbed to the sensor when used for longer times. Secondly, the effective surface area of the sensors should be optimized for detecting NTs. Miniaturizing the sensors by using nanofiber material during sensor design will increase the effective contact surface area between the sensors and the analytes being tested. Finally, the effect of crosstalk between the many species of NTs should be studied because many NTs coexist in the brain environment at varying concentration levels. Although the selected concentrations of NTs were close to the physiological levels, they may not reflect the reality of the internal environment of the brain where several NTs coexist at different volumes and concentrations, exhibiting different mutual interactions. Better sensors considering the solutions proposed in this section are under development.

## 8. Conclusions

In this work, we developed a novel automatic approach for estimating the concentrations of DA and SE in in vitro mixtures, reducing the procedural complexity of the estimation. DA and SE were simultaneously and accurately detected, and their concentrations were predicted with the two proposed pattern recognition techniques, PCA-GPR and PLS-GPR, in in vitro mixtures using differential pulse voltammetry and conventional glassy carbon electrodes. The results are summarized in Table 2 and Table 3. Further, the feature subsets serving as predictors for the NT concentration levels were determined and reduced, thereby reducing the complexity of estimating the NTs. Using PCA-GPR on the reduced feature subsets, the maximum average training and testing accuracies for detecting and predicting DA alone were both 86.73%, 87.83% and 87.93% for SE alone, respectively, and 97.41% and 97.4% for simultaneous DA–SE, respectively. We thus believe that the reduced system complexity for NT recognition will be of genuine application value in medical therapies like closed-loop deep brain stimulation for the treatment of Parkinson’s disease or Alzheimer’s disease. Nevertheless, the accuracies obtained were negatively influenced by some issues linked to the sensors like the passivation of the sensors, crosstalk, sensor material, and sensors’ contact surface area with the NTs. Solving these issues as well as optimizing the experimental conditions are avenues for improving the results in future work.

## Figures and Tables

**Table 1:** Training sets and testing set from each concentration combination.

Experiment Number	Training Sets (with 10-Fold Cross-Validation)	Testing Set (Independent Set)
Exp 1	1 and 2	3
Exp 2	1 and 3	2
Exp 3	2 and 3	1

**Table 2:** Result summary for prediction accuracies and R^2^ values for PCA-GPR.

Dataset Feature Voltages	Training and Testing	Accuracy	R^2^ Values
DA			
All features −0.16 V to 0.88 V	TR	87.6	0.75
	TE	87.6	0.77
OPW features 0 V to 0.52 V	TR	86.2	0.72
	TE	87.6	0.78
SE			
All features −0.16 V to 0.88 V	TR	88.0	0.72
	TE	88.1	0.74
OPW features 0 V to 0.52 V	TR	87.3	0.70
	TE	87.9	0.74
DA–SE			
All features −0.16 V to 0.88 V	TR	96.7	0.76
	TE	96.7	0.77
OPW features 0 V to 0.52 V	TR	96.4	0.73
	TE	96.5	0.77

**Table 3:** Result summary for prediction accuracies and R^2^ values for PLS-GPR.

Dataset Feature Voltages	Training and Testing	Accuracy	R^2^ Values
DA			
All features −0.16 V to 0.88 V	TR	88.2	0.77
	TE	87.3	0.77
OPW features 0 V to 0.52 V	TR	87.7	0.76
	TE	86.8	0.77
SE			
All features −0.16 V to 0.88 V	TR	88.5	0.71
	TE	83.8	0.58
OPW features 0 V to 0.52 V	TR	87.7	0.78
	TE	85.0	0.65
DA–SE			
All features −0.16 V to 0.88 V	TR	96.8	0.77
	TE	95.1	0.60
OPW features 0 V to 0.52 V	TR	96.8	0.78
	TE	95.4	0.66

**Table 4:** Result summary for prediction accuracies and R^2^ values for PCA-GPR with reduced feature subsets.

NT	Columns of Subset from Original Dataset	Training and Testing	Accuracy	R^2^ Value
DA	9, 8, 10, 11, 7	TR TE	86.73 86.76	0.75 0.756
SE	13, 15, 16, 14	TR TE	87.83 87.93	0.74 0.743
DA–SE	13, 15, 14, 12, 16	TR TE	97.41 97.4	0.75 0.743

**Table 5:** Prediction accuracies and R^2^ values for PCA-GPR with reduced feature subsets with data from Sazanova et al. [1].

NT	Columns of Subset from Original Dataset	Training and Testing	Accuracy	R^2^ Value
DA	9, 8, 10, 11, 7	TR TE	83.73 82.76	0.74 0.75
SE	13, 15, 16, 14	TR TE	86.83 87.93	0.76 0.73
DA–SE	13, 15, 14, 12, 16	TR TE	95.42 96.3	0.74 0.73

## Data Availability

The data and code supporting this study will be made available by the corresponding author upon reasonable request.
